# New objective simple evaluation methods of amyloid PET/CT using whole-brain histogram and Top20%-Map

**DOI:** 10.1007/s12149-024-01956-y

**Published:** 2024-06-22

**Authors:** Chio Okuyama, Tatsuya Higashi, Koichi Ishizu, Naoya Oishi, Kuninori Kusano, Miki Ito, Shinya Kagawa, Tomoko Okina, Norio Suzuki, Hiroshi Hasegawa, Yasuhiro Nagahama, Hiroyuki Watanabe, Masahiro Ono, Hiroshi Yamauchi

**Affiliations:** 1 Clinical Research Center, Shiga General Hospital, Moriyama, Japan; 2 Department of Molecular Imaging and Theranostics, National Institute of Quantum Science and Technology, Chiba, Japan; 3 Human Health Sciences, Graduate School of Medicine, Kyoto University, Kyoto, Japan; 4 Human Brain Research Center, Graduate School of Medicine, Kyoto University, Kyoto, Japan; 5 Department of Radiology, Shiga General Hospital, Moriyama, Japan; 6 Department of Neurology, Shiga General Hospital, Moriyama, Japan; 7 Department of Psychiatry and Neurology, Kawasaki Memorial Hospital, Kawasaki, Japan; 8 Department of Patho-Fundamental Bioanalysis, Graduate School of Pharmaceutical Sciences, Kyoto University, Kyoto, Japan; 9 Department of Psychiatry, Graduate School of Medicine, Kyoto University, Kyoto, Japan

**Keywords:** Amyloid PET, Objective evaluation, Histogram, Alzheimer’s disease, Skewness

## Abstract

**Objective:**

This study aims to assess the utility of newly developed objective methods for the evaluation of intracranial abnormal amyloid deposition using PET/CT histogram without use of cortical ROI analyses.

**Methods:**

Twenty-five healthy volunteers (HV) and 38 patients with diagnosed or suspected dementia who had undergone ^18^F-FPYBF-2 PET/CT were retrospectively included in this study. Out of them, ^11^C-PiB PET/CT had been also performed in 13 subjects. In addition to the conventional methods, namely visual judgment and quantitative analyses using composed standardized uptake value ratio (comSUVR), the PET images were also evaluated by the following new parameters: the skewness and the mode-to-mean ratio (MMR) obtained from the histogram of the brain parenchyma; Top20%-map highlights the areas with high tracer accumulation occupying 20% volume of the total brain parenchymal on the individual’s CT images. We evaluated the utility of the new methods using histogram compared with the visual assessment and comSUVR. The results of these new methods between ^18^F-FPYBF-2 and ^11^C-PiB were also compared in 13 subjects.

**Results:**

In visual analysis, 32, 9, and 22 subjects showed negative, border, and positive results, and composed SUVR in each group were 1.11 ± 0.06, 1.20 ± 0.13, and 1.48 ± 0.18 (*p* < 0.0001), respectively. Visually positive subjects showed significantly low skewness and high MMR (*p* < 0.0001), and the Top20%-Map showed the presence or absence of abnormal deposits clearly. In comparison between the two tracers, visual evaluation was all consistent, and the ComSUVR, the skewness, the MMR showed significant good correlation. The Top20%-Maps showed similar pattern.

**Conclusions:**

Our new methods using the histogram of the brain parenchymal accumulation are simple and suitable for clinical practice of amyloid PET, and Top20%-Map on the individual’s brain CT can be of great help for the visual assessment.

## Introduction

Two characteristic neuropathological changes in Alzheimer’s disease (AD) are the extracellular amyloid senile plaques and the intracellular neurofibrillary tangles (NFTs) [1, 2]. According to the well-known amyloid cascade hypothesis, the intracranial amyloid beta (Aβ) protein deposition is responsible for the pathogenesis of AD [3]. Aβ molecules tend to aggregate, forming oligomer, soluble protofibril, and finally insoluble mature fibrils known as senile plaques [4, 5]. On January 6, 2023, the U.S. Food and Drug Administration (FDA) approved lecanemab, humanized monoclonal antibody that targets soluble Aβ protofibrils for the treatment of Alzheimer’s disease (AD). After that, lecanemab has been approved in many countries one after another, and the treatment of MCI and early dementia caused by AD has now reached a major turning point. The deposition of Aβ is, therefore, one of the main targets of in vivo imaging, and amyloid PET has been an accepted diagnostic imaging tool for AD.

Carbon-11 labeled Pittsburgh compound B (PiB) was the first developed Aβ PET tracer [6], and it has been considered reliable to evaluate the intracranial amyloid deposition and used widely as a research tool [7]. However, the short half-life of ^11^C limits the feasibility, so several ^18^F-labeled Aβ tracers with longer half-life, such as ^18^F-flutemetamol [8], ^18^F-florbetapir [9], or ^18^F-florbetaben [10], have been developed for commercial clinical practice. 5-(5-(2-(2-(2-^18^F-Fluoroethoxy)-ethoxy)-ethoxy)-benzofuran-2-yl)-*N*-methylpyridin-2-amine (^18^F-FPYBF-2) is one of the new ^18^F-labeled amyloid tracers which our coauthors developed [11]. So far, using this tracer, we published the first-in-human data of the biodistribution and radiation dosimetry assessment [12] and clinical utility to evaluate Alzheimer and related diseases [13], and amyloid deposition in patients with chronic focal or diffuse traumatic brain injury [14]. Our prior study showed its comparable diagnostic ability of ^18^F-FPYBF-2 with ^11^C-PiB [13].

Visual evaluation methods using polarized judgment of positive or negative have been used for clinical situation [15]. However, amyloid deposition progresses gradually in time, and cannot always be clearly separated into positive and negative. Quantitative evaluation with standardized uptake value ratio (SUVR), the accumulation ratio with reference sites such as the cerebellar cortex, is, thus, used in many research evaluations and as a quantitative indicator of individual accumulation. However, placing regions of interest (ROIs) in the cortex manually is not practical because of the intricately convoluted gyri and the thin cortical gray matter. Therefore, automatic methods with ROI templates after converting patient's images to a standard brain are usually used.

However, the shape of a patient’s brain is not same as the standard brain, and the automatically placed ROIs do not exactly represent the individual's anatomical location. For example, the posterior cingulate gyrus, where early pathological changes are known [16, 17], is adjacent to the corpus callosum with high tracer accumulation. Moreover, enlarged ventricles and the presence of subdural effusion often cause mis-registration that affects quantitative evaluation by automated ROI analysis. For such reasons, visual interpretation is considered essential for individual cases in daily clinical practice.

In order to establish a widely usable objectively quantifying method for Aβ tracer, we have developed a new method using a simple histogram from PET/CT data, without converting to a standard brain or using ROI analyses. We have also devised a color-mapping method to aid for visual diagnosis by clearly depicting tracer accumulation areas on the individuals’ brain CT images. The aim of this study is to evaluate the utility of these newly developed evaluation methods for amyloid PET/CT using the ^11^C-PiB and ^18^F-FPYBF-2 image data from our previous studies in a retrospective manner.

## Materials and methods

This study was approved by our institutional review boards, the Human Study Committee (approved on Sept 7th, 2021) and conducted in accordance with the Declaration of Helsinki. In this study, two major evaluations were included: A, the utility of histogram analyses using ^18^F-FPYBF-2 comparing with visual evaluation and conventional ROI analyses using SUVR; B, comparison of the histograms analyses between two different PET tracers (^18^F-FPYBF-2 and ^11^C-PiB). For each purpose, we used the PET/CT data obtained in our previous studies: the first-in-human study using ^18^F-FPYBF-2 for subjects with cognitive disorders or healthy volunteers, and the comparative study between ^18^F-FPYBF-2 and ^11^C-PiB PET [13]. These PET studies were approved by our institutional review boards, the Human Study Committee (approved on Sep. 25, 2013, and on Jan. 18, 2016). Each subject gave a written informed consent for the previous studies. Because of the retrospective study design of this study and the way of using anonymized subject data, requirement for additional informed consent was waived for the purpose of this additional analysis using histogram for the patients who had undergone the PET studies previously.

### Subjects

The subjects were cognitively normal healthy volunteers (HV) and patients with suspected or diagnosed dementia, who were the subjects for our previous first-in-human studies using ^18^F-FPYBF2 [13], Out of all the subjects (*n* = 116) of the previous studies, those who had undergone PET/CT examination were included in this study (*n* = 63). The cases examined by a dedicated PET machine were excluded (*n* = 53).

A. Evaluation of Histogram analyses using ^18^F-FPYBF-2 PET/CT

For evaluation of the utility of the histogram, the data of ^18^F-FPYBF-2 PET/CT performed in 25 HV and 38 patients with diagnosed or suspected dementia or related diseases (27 men and 36 women; mean age: 68.7 ± 12.4 (range: 40–88) years old) were used. Of the 38 patients, 15 had been clinically diagnosed as having AD, 17 mild cognitive impairment (MCI), and 6 other disease including dementia of Lewy body, amyloid angiopathy and vascular dementia, and front-temporal lobe dementia (Others).

B. Comparison of the histogram analyses between two different tracers

Thirteen subjects, 7 men and 6 women; mean age 70.5 ± 11.7 years old, included 2 HV, and 11 patients of MCI (*n* = 3), AD (*n* = 4), and others (*n* = 4). All these subjects underwent PET/CT studies using two different tracers: ^18^F-FPYBF-2 and ^11^C-PiB. The intervals of the two PET/CT studies were within 2 weeks for patients with dementia or MCI and half a year for HV.

### PET/CT examination

Both ^18^F-FPYBF-2 and ^11^C-PiB were synthesized in-house. The radiosynthesis of ^18^F-FPYBF-2 and ^11^C-PiB was performed using modification of the methods described elsewhere [11, 18] with a hybrid synthesizer and cassette-type multipurpose automatic synthesizer module (JFE Engineering Corporation, Japan).

PET/CT scans were performed by a whole-body PET/CT scanner, Siemens TruePoint Biograph 16 (Siemens/CTI, Erlangen, Germany). Static head PET image was acquired 50–70 min after the intravenous injection of ^18^F-FPYBF-2 (3.7–4.0 MBq/kg) and ^11^C-PiB (7.4–9.0 MBq/kg), respectively. Matrix size was 256 × 256, and acquisition data were reconstructed using the back projection reconstruction, and images were blurred to 6.0 mm full width at half-maximum in the transaxial direction using a Gaussian filter.

A CT scan was performed for attenuation correction (tube voltage, 130 kV; tube current, 30 mA; tube rotation time, 0.6 s per rotation; pitch, 1.0). The CT data were resized from a 336 × 336 matrix to a 256 × 256 matrix to match the PET data and construct the CT-based transmission maps for attenuation correction of the PET data with a post-reconstruction Gaussian filter (5 mm FWHM). The reconstructed voxel size of the PET/CT images was 2.3 × 2.3 × 4 mm.

### Data analyses

PET images are evaluated by two conventional methods: visual evaluation and quantitative analysis using automated ROI, and two new methods: whole-brain histogram parameter analyses and the evaluation of the color map named Top20%-Map.

#### Visual evaluation of PET images

The axial coronal and sagittal PET images were evaluated using PRISM color and gray scale expressed with the SUVR display (range: 0–3) with the reference on the cerebellar cortex. For the gray scale images, we also evaluated by freely adjusting the gradation of the DICOM image on the image viewer.

The PET images were visually evaluated by three experienced nuclear medicine experts (C.O., T.H., and K.I.) who met at least one of the following conditions regarding the experiences for the diagnosis of amyloid PET: (1) with the experience of diagnosing more than 100 cases of amyloid PET, (2) with the board certification for the interpretation of amyloid PET by Japanese society of Nuclear Medicine. Without any clinical information of the cases, each reviewer separately interpreted the PET images and classified them into three scores: 0, no abnormal amyloid deposition; 1, equivocal finding; 2, abnormal amyloid deposition. The scores of the three reviewers were summed up, and the final visual estimation was determined as below: negative, the summed score was 0 or 1; border, 2–4; positive, 5 or 6.

#### Quantitative analysis using automated ROI

For the quantitative evaluation of the amyloid deposition, SUV of each region (rSUVR) was measured using the cerebellar cortex as a reference [19, 20]. To obtain rSUVR, we performed automated ROI analysis using the automated anatomical labeling atlas (AAL) [21] as described in our previous manuscript [13].

The difference from our previous studies was using CT images obtained PET/CT to convert it into a standard brain. CT images were spatially normalized to the in-house CT template and all AAL ROIs in the standard space were inversely transformed to individual spaces by the inverse deformation field.

The ROIs on the cerebellar cortex were combined and used as a reference region to create SUVR images [22]. Mean rSUVR values within 90 anatomical ROIs in both hemispheres were calculated by an in-house Matlab script. Then, as a value for abnormal cortical amyloid deposition of each subject, the mean rSUVR value within the frontal, posterior cingulate, precuneus, parietal and lateral temporal cortical regions was calculated as composed SUVR (ComSUVR) [23].

#### Whole brain histogram parameter analysis and Top20%-Map

**Fig. 1:**
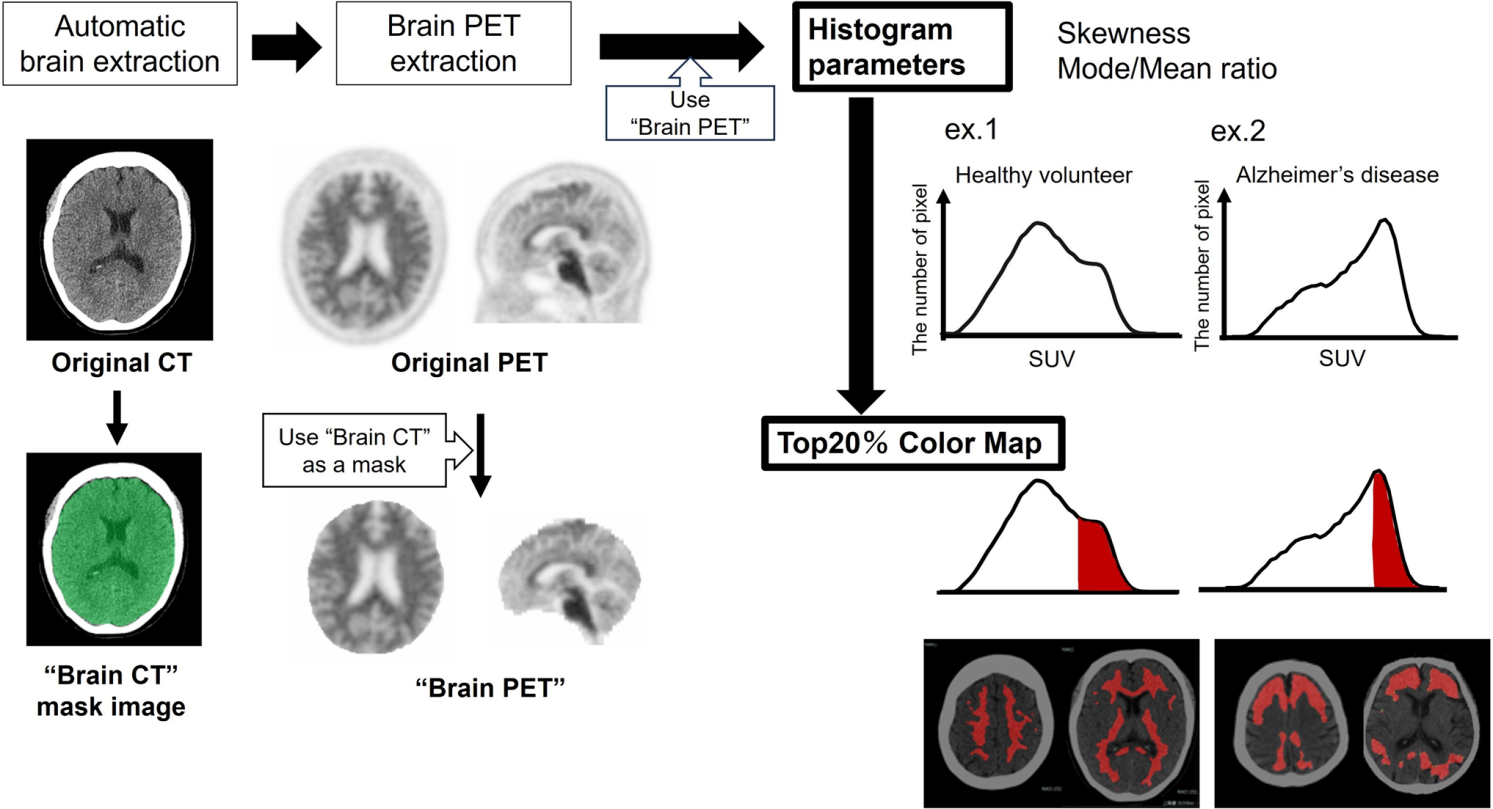
The overview of the process of creating whole-brain histogram and Top20%-Map

Figure 1 shows an overview of the process creating histogram and “Top20%-Map”. For the extraction of whole-brain histogram and the creation of the map, a commercially available imaging workstations, CT volume analyzer (SYNAPSE VINCENT medical imaging system; FUJIFILM Medical Tokyo, Japan) was used.

To create whole-brain histogram, we used the application named “multi-3D” that can deploy multiple modalities or series to the viewer simultaneously and perform individual processing. At first, using the automatic brain extraction algorithm implemented in the volume analyzer, the skull and the soft tissue of the head were removed automatically on the CT data to extract “Brain CT.” In this process, whole brain including the brain stem is extracted automatically. Subsequently, “Brain PET” was extracted by applying “Brain CT” to the PET images as a mask image. On the extracted "Brain PET" image displayed in 3D, a spherical VOI including the whole brain was set to obtain the whole-brain histogram of the SUV with a bin size of 0.05. The process of automated brain-PET extraction and the creation of its histogram from one subject took about 5 min.

Then, after removing the data with no counts corresponding to the data in the ventricle or outside the brain in the VOI, we exported the histogram data as a CSV file to a statistical software JMP®14.3 (SAS Institute Inc., Cary, NC, USA) to extract parameters including skewness, mean, and mode values from the histogram and the mode/mean ratio (MMR) was calculated according to the following formula:

$$\text{MMR}=\frac{\text{mode value}}{\text{mean value}}.$$

Subsequently, using the fusion algorithm implemented in the volume analyzer, high-accumulation area corresponding to 20% volume of the whole-brain parenchyma was colored on the subject’s CT images. Creating the Top20%-Map includes the following three steps: 1st, Measure of the brain volume; 2nd, Scaling of the top 20% SUV; 3rd, Color mapping on the CT images.

1. First step: Measure of the brain volume

After overlaying “Brain PET” data on the CT images, a spherical large ROI that includes the entire brain was set. The minimum value of the PET display was gradually raised from zero until tracer accumulation was visually invisible in the lateral ventricle so as to calculate the volume of whole-brain parenchyma.

(2) Second step: Scaling of the top 20% SUV

After determining the volume of the whole-brain parenchyma, the minimum value of the PET display was increased to determine the top20% SUV at which only 20% of the total volume with high uptake was displayed.

(3) Third step: Color mapping on the CT images

On the fused images, the top20% SUV is input to both the maximum and minimum values of the PET display. In this way, high-accumulation areas that account for 20% of the total volume are highlighted on the individual’s CT images. These steps creating Top20%-Map for one subject took about 3 min.

(4) Evaluation of the Top20%-Map

**Fig. 2:**
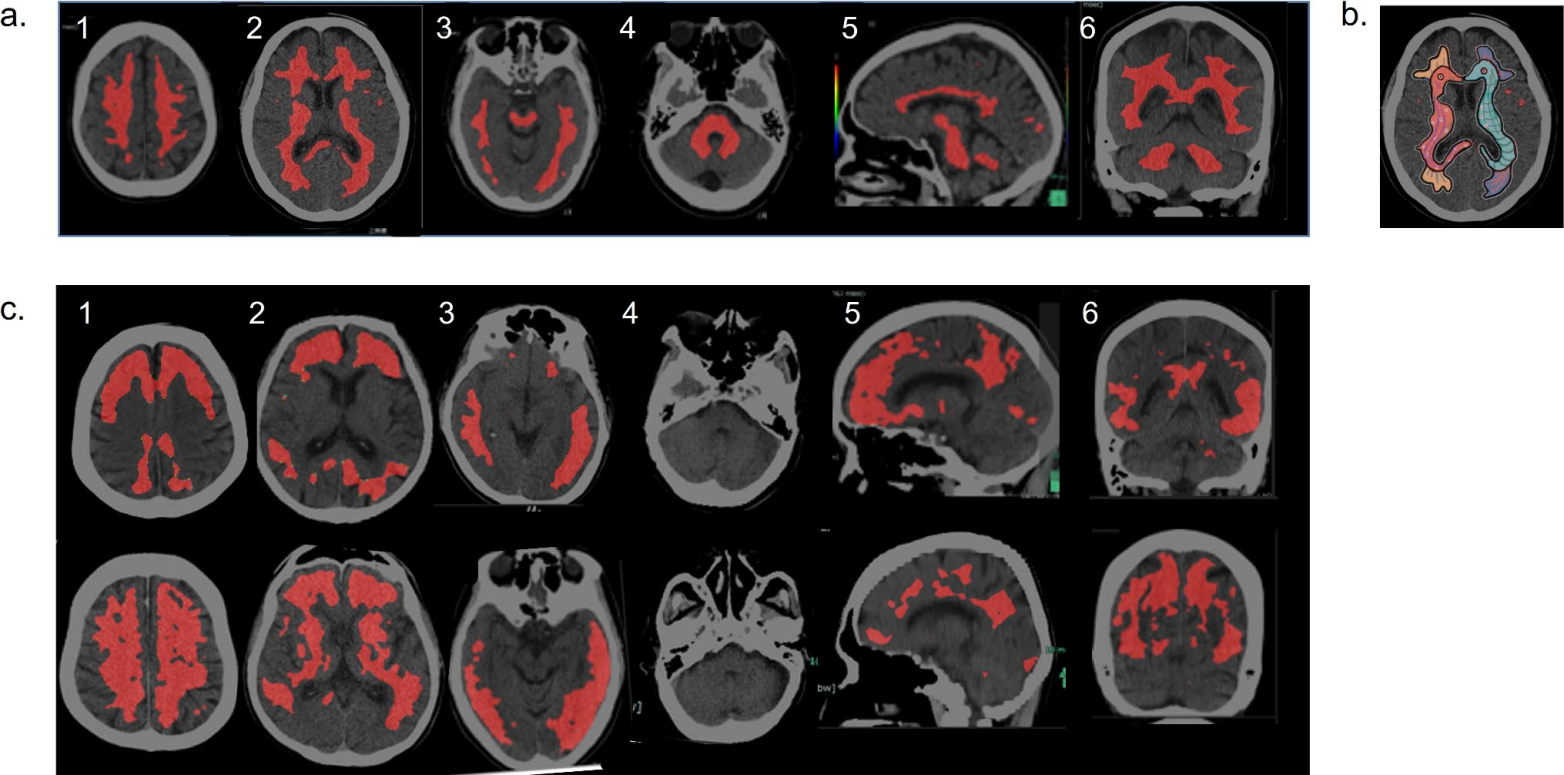
Findings of Top20%-Map. Normal visualization of the deep white matter/brain stem and cerebellar peduncle/corpus callosum are evaluated in the level 1, 2, 3/3, 4, 5, 6/2, 5, 6. **a** In Normal map on the axial image of the level of basal ganglia, the highlighted area looks like two seahorses kissing (**b**). Abnormal cerebral cortical uptake/ posterior cingulate and precuneus were evaluated in 1, 2, 3, 6/1, 5, 6 (**c**)

Figure 2a shows the representative Top20%-Map of a HV without abnormal amyloid deposition. Deep white matter, corpus callosum, posterior limbs in the internal capsule, brain stem and cerebellar white matter are visualized. On the axial image of the levels of the basal ganglia, the highlighted area seems like two kissing seahorses with pointed horns and long beaks (Fig. 2b). When kissing seahorses, brainstem and cerebellar peduncles were all observed, the maps were judged as normal map. On the other hand, abnormal map had the both following two findings: (1) highlighted cerebral cortex and/or posterior cingulate gyrus, (2) invisible seahorse or no-colored cerebellar peduncle and brainstem (Fig. 2c). The cases with one of the following findings were determined to be a borderline map: (1) the conditions for normal map are met, but also the cortex and cingulate gyrus are partially highlighted; (2) invisible seahorse or brain stem without highlighted cortex and cingulate gyrus.

### Statistical analyses

All numeric values were expressed as mean ± standard deviation (SD). All statistical analyses were performed using JMP®15.3.0 statistical software (SAS Institute Japan, Tokyo) with *p* values < 0.05 denoting statistical significance. A comparison between each group was analyzed by the Wilcoxon test or the Kruskal–Wallis analysis for unpaired data. To compare the categorical data for the visual evaluation, we used chi-square tests. For the comparison of the ComSUVR, skewness, and MMR between ^18^F-FPYBF2 and ^11^C-PiB, Spearman’s rank correlation test and paired Wilcoxon signed-rank sum test were performed.

## Results

### Histogram analyses using ^18^F-FPYBF-2 PET/CT

#### Automated ROI evaluation and Visual evaluation according to the clinical diagnosis

**Fig. 3:**
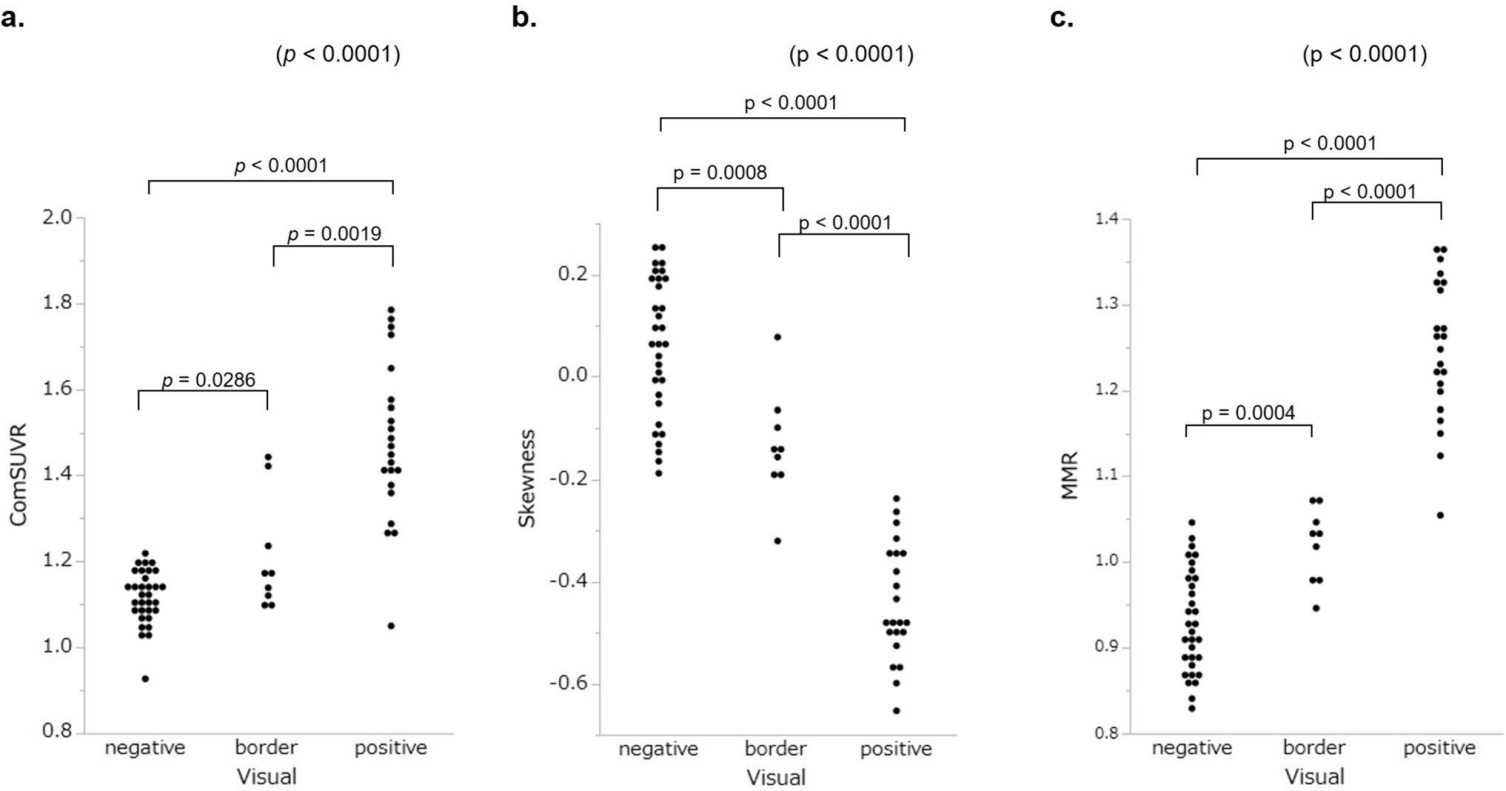
ComSUVR, skewness and MMR according to the visual evaluation

In the visual evaluation of 25 HV, negative, border, and positive results were observed in 23, 1, and 1 subjects, respectively. In 15 with clinically diagnosed AD cases, 18 MCI and 6 others, negative/border/positive findings were observed in 1/2/12, 6/5/6, and 2/1/3, respectively. AD group showed significantly high ComSUVR (1.50 ± 0.19) compared with HV (1.12 ± 0.06), MCI (1.25 ± 0.16), and Others (1.18 ± 0.24) (*p* < 0.0001). For the purpose to evaluate the utility of our new analyses, we used the results of visual evaluation as the reference regardless of the clinical diagnosis since the clinical diagnosis is not always right and sometimes corrected after amyloid PET. ComSUVR were 1.11 ± 0.06, 1.20 ± 0.13, 1.48 ± 0.18 in the groups with visually negative, border and positive findings, respectively (*p* < 0.0001) (Fig. 3a).

#### Histogram parameters according to the visual evaluation

**Fig. 4:**
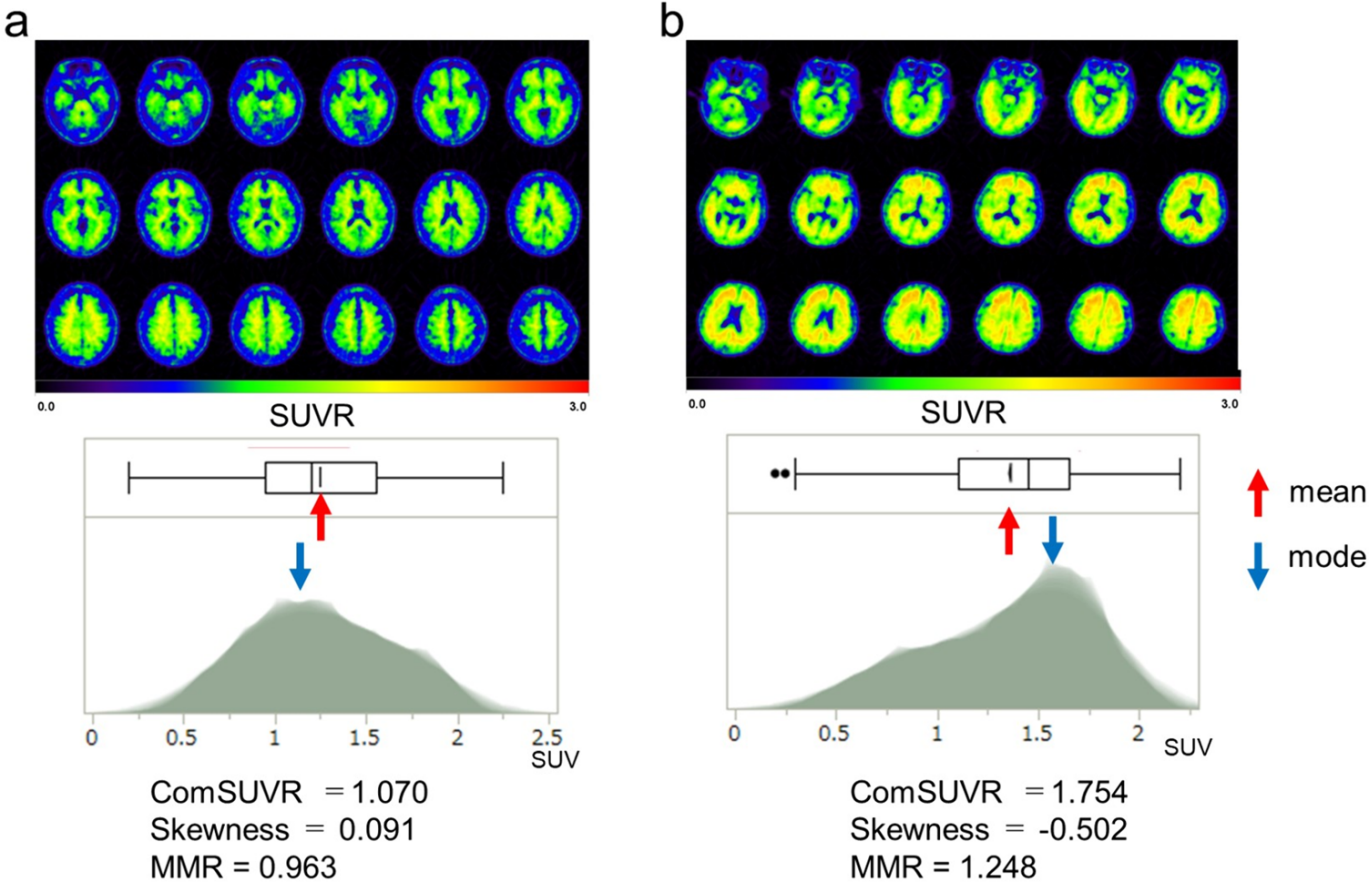
Two examples of ^18^F-FPYBF-2 PET images and the histogram: **a** healthy volunteer; **b** a case with Alzheimer’s disease

**Fig. 5:**
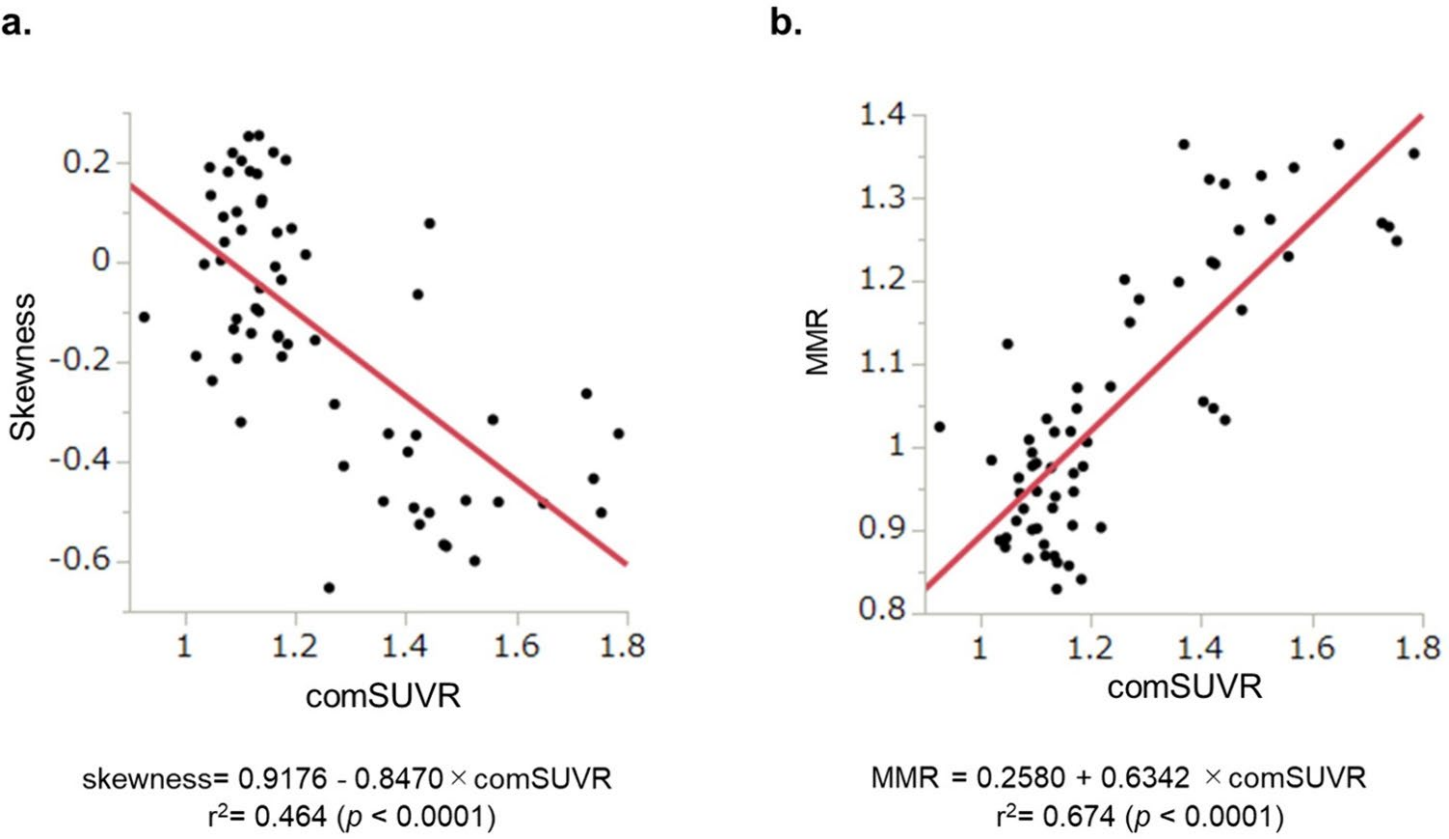
The relationships between comSUVR and skewness (**a**) and between comSUVR and MMR. **a** Showed a significantly mild negative correlation, and **b** showed a significantly positive correlation

Figure 3b and c demonstrates the skewness and the MMR according to the visual evaluation. Patients with visually positive results showed significantly low skewness and high MMR (*p* < 0.0001). Figure 4 shows the representative PET images and the histograms in a HV subject (a) and an AD patient (b). Skewness showed significantly negative correlation and MMR showed strong positive correlation with ComSUVR (*p* < 0.0001) (Fig. 5).

#### Top20%-Map

Table 1 demonstrates the results of Top20%-Map evaluation according to the visual evaluation. All cases with visually positive results showed abnormal map, and 30 of 32 cases with visually negative results showed normal map. Two cases with visually negative image but borderline map had suspected old vascular lesions in the deep white matter that probably made seahorse invisible. In two cases with visually border image but abnormal map depicted localized cortical accumulation only in the orbital and posterior cingulate gyri.

**Table 1:** The evaluation of Top20%-Map according to the visual evaluation

	Top20%-Map evaluation			
	Normal map	Borderline map	Abnormal map	
Visual evaluation				
Negative	30	2	0	32
Border	3	4	2	9
Positive	0	0	22	22
	33	6	24	

### Comparison between ^18^F-FPYBF-2 and ^11^C-PiB

**Fig. 6:**
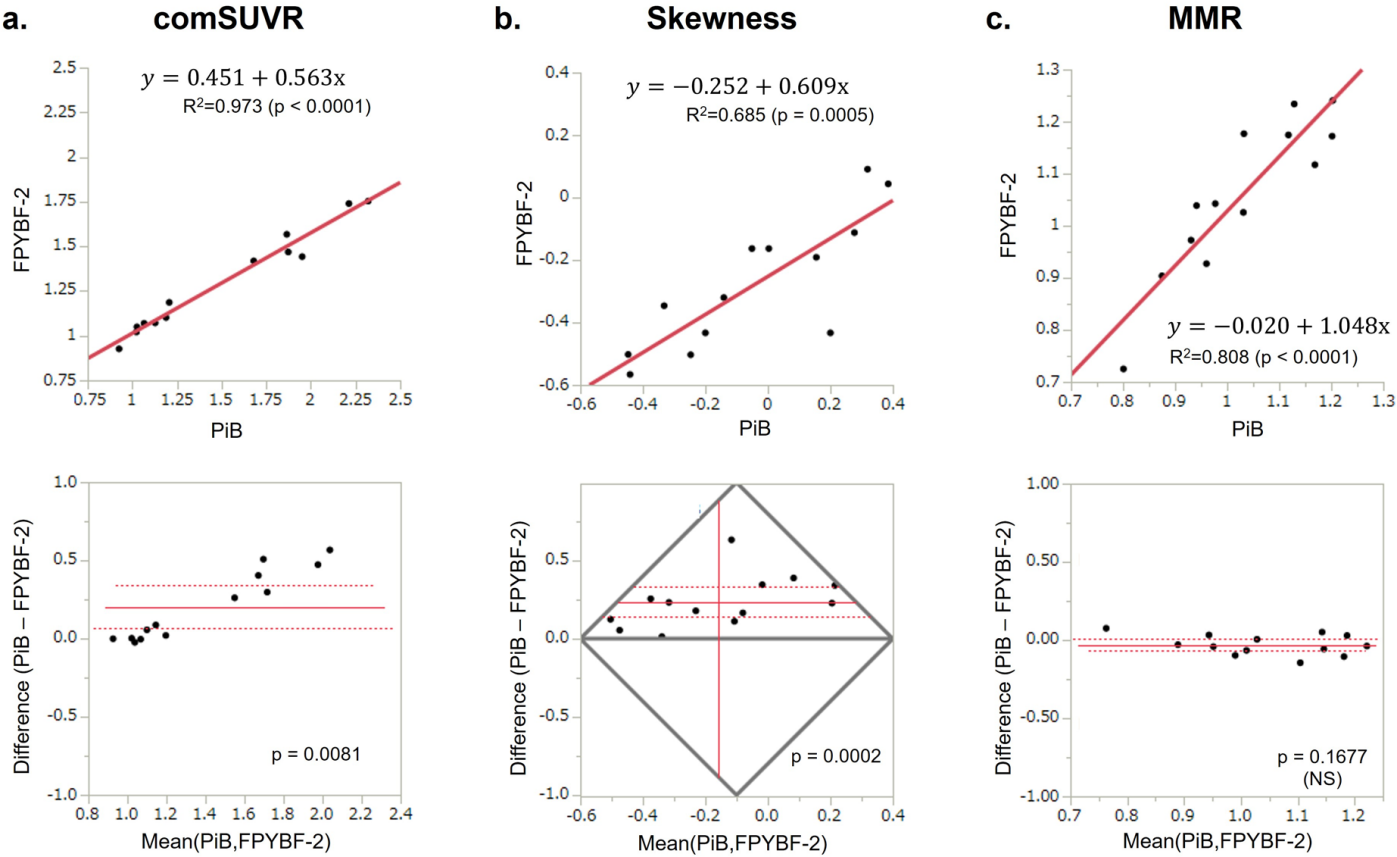
The results of comSUVR (**a**), skewness (**b**) and MMR (**c**) between ^11^C-PiB and ^18^F-FPYBF-2 (upper column: the relationship, lower column: Bland–Altman plot). Each parameter showed a good correlation between the two tracers. MMR showed no significant difference between the tracers, while the comSUVR and skewness of ^11^C-PiB were significantly higher than those of ^18^F-FPYBF-2

The results of visual evaluation were all consistent between the two different tracers: 5 negative, 1 border, and 7 positive. Figure 6 demonstrates the comparison of ComSUVR, skewness, and MMR between them. ComSUVR showed very strong correlation (*r*^2^ = 0.973, *p* < 0.0001), and both skewness and MMR showed positive correlation (*r*^2^ = 0.685, *p* < 0.0001, and *r*^2^ = 0.808, *p* < 0.0001, respectively), as well. Although^11^C-PiB showed significantly higher values of ComSUVR and skewness than ^18^FPYBF-2, especially in visually positive cases, MMR showed no significant difference between the two tracers.

**Fig. 7:**
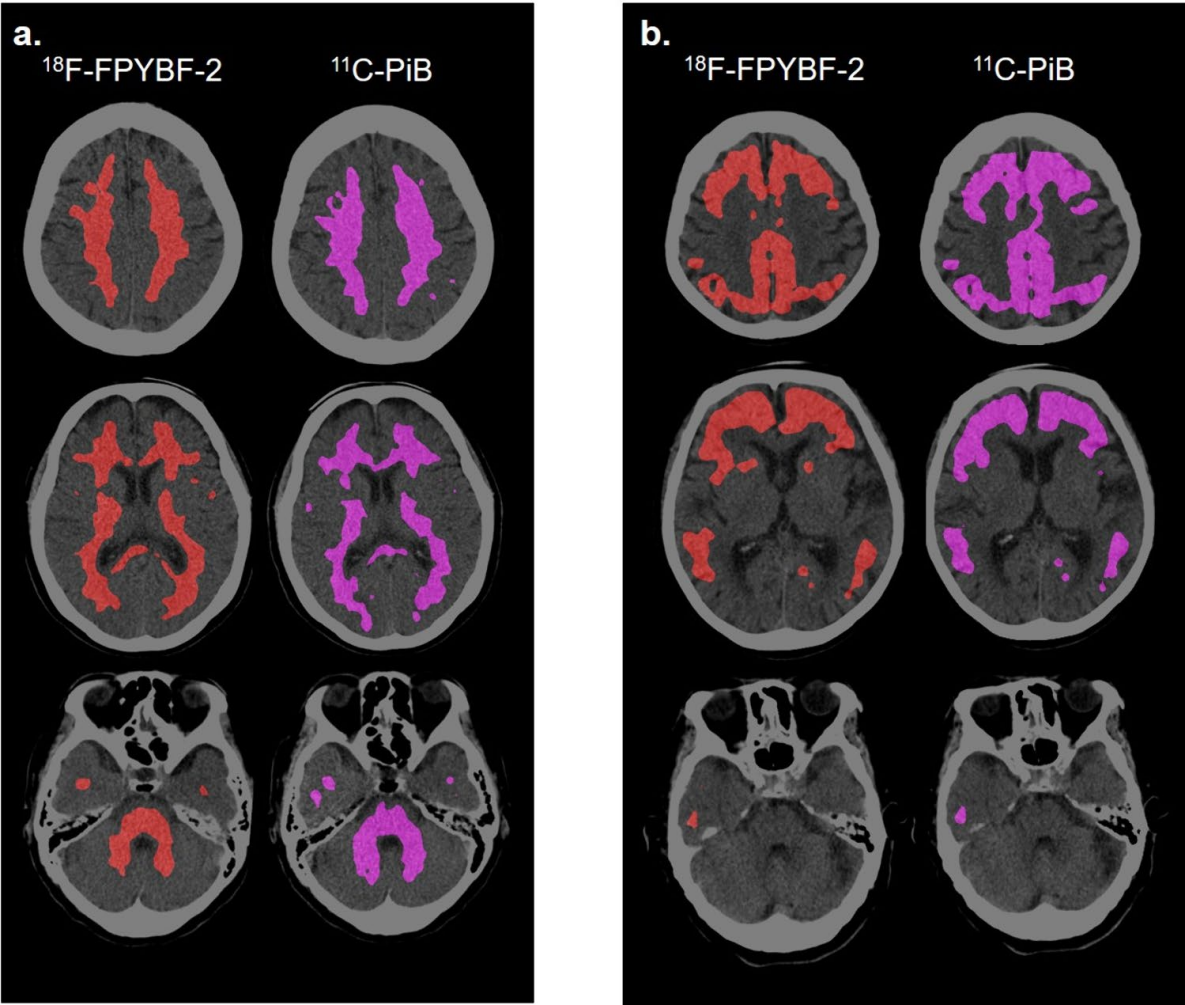
The comparison of Top20%-Map in three cases: **a** normal map in a healthy volunteer; **b** abnormal map in a patient with Alzheimer’s disease

Five, two and six subjects showed normal, borderline, and abnormal map, consistently in both tracers. Figure 7 shows the Top20%-Maps of the two tracers in 3 subjects: a, normal map; b abnormal map. The maps of the two different tracers were almost indistinguishable in each subject.

## Discussion

Our data suggested that these new histogram evaluation methods are simple, objective, and comparable with conventional visual evaluation and semi-quantitative ROI analysis using SUVR. In the whole-brain histogram of visually amyloid positive cases, negative skewness means data deviation to the right, and high mode/mean ratio (MMR) was characteristically observed. These parameters showed good correlation with the ComSUVR. Moreover, the Top20%-Maps provided visually informative amyloid deposition maps on the individual’s brain CT images, not on a standard brain such as Talairach. Comparative analysis of ^11^C-PiB and ^18^F-FPYBF-2 showed a significant correlation in each parameter, and the Top20%-Maps of both tracers showed similar images.

Since amyloid tracer shows high physiological off-target accumulation in the white matter, evaluating abnormal amyloid deposition in the cerebral gray matter needs careful attention. Moreover, tracer distribution rate to the brain is known to be small at around 1–2% of the total dose [12, 24], which results in a difficult situation for the quantitative analysis using SUV within a very narrow range. Therefore, a relative SUV ratio (SUVR) to a reference part is usually used for the quantitative assessment of brain amyloid PET. The cerebellar cortex, which is known to have little amyloid deposition [19, 20], is often used as a reference. White matter, pons, or corpus callosum is sometimes used as the reference area [25–27] because the cerebellar cortex is not a valid reference region in case with significant amyloid deposition in the cerebellar cortex, such as familial AD and severe AD [28], cerebral amyloid angiopathy, or a certain type of systemic amyloidosis [29, 30].

Wherever the reference is set, it is not easy to correctly place the ROIs at thin and complexly folded cerebral cortex, and usually automatic ROI templates are applied after converting the patient's brain into a standard brain. Such methods can reduce inter-observer or inter-operator differences compared to manual ROI setting [31], but the difference of individual’s brain morphology and incorrect insertion of white matter with relatively high accumulation in the cortical ROI would be problematic.

Our new method using a histogram of the tracer accumulation of the whole brain after removing skull and skin is completely different from the conventional ROI analysis using SUVR. The degree of cortical abnormal uptake can be expressed easily by the shape of histogram and its parameters. This method is simple and can be performed using a 3D-Fusion analyzer in a short time. For creating the histograms, we used SUV that can be easily obtained through PET examination without complicated steps to set ROIs. Theoretically, SUVR or even the original PET count data can provide the similar histogram and the parameters.

Several amyloid PET tracers have been developed and comparative studies between ^11^C-PiB and each tracer have been performed [32–38]. Although many previous researches revealed that the almost concordant diagnostic abilities between different tracers [32–38], there are some differences in the distribution among tracers [39, 40], and most ^18^F-labeled tracers are known to show a narrow dynamic range than that of ^11^C-PiB [9, 38, 41, 42]. In this decade, the centroid scale has become popular as a method to resolve the differences between tracers [43]. Centiloid values are calculated by converting the ComSUVR of each amyloid PET image to the ComSUVR obtained using ^11^C-PiB and standardizing to a scale from 0 to 100 regardless of tracers. Centiloid scale is, thus, now expected to solve the problem of the polarized visual judgment in the cases of positive/negative border area, but its potential problems caused using ROIs remain unsolved.

Our histogram-based method with some parameters and Top20%-Map using anatomical information of the individuals’ brain is simple and able to evaluate the degree of amyloid accumulation without making a polarized judgment. Similar to Centiloid scale, the new methods can be used in clinical situation for evaluating minor deposits in the cases of border area, and for evaluating the progression or the treatment effect after a certain period of time. Besides numerical parameters, such as skewness and MMR, the shape of the histogram itself and Top20%-Map can provide visually comprehensible information.

The limitation of the present study should be addressed. First, the detailed mechanism by which skewness is negative and MMR high in cases with abnormal amyloid deposition has not been elucidated. It is still unknown whether our new methods can reduce the effects of white matter with high non-specific accumulation or not. If we could extract and analyze histograms of cerebral cortex and white matter separately, it would be possible to make an evaluation that reflects the pathological image of abnormal amyloid deposition. Second, it is also unclear whether the new methods will work for other tracers either. Although there was no significant difference in MMR values between ^18^F-FPYBF-2 and ^11^C-PiB, the values of ComSUVR and skewness showed significant differences between them. Further study focusing the different characteristic of other tracers would be needed. Regarding the Top20%-Map, we defined the threshold volume as 20% in this study so that only the off-target area is highlighted in negative cases. This figure, 20%, may have to be reconsidered when being applied for other tracers.

In conclusion, this study showed that our new histogram analysis and Top20%-Map that do not need ROI analysis are useful method for evaluating amyloid deposition. The objective parameters we proposed can be calculated using a versatile 3D-image analyzer, without using specific image analysis software or Matlab. The Top20%-Map directly reflects the shape of the patient's brain rather than the standard brain, so that it is easy to evaluate the individual uptake area correctly. The simplicity of this method is suitable for evaluation in daily clinical practice to determine treatment strategies for patients with suspected AD.

## Data Availability

The use of datasets generated and analyzed during the current study is approved for paticipated institutions by Ethics Committee. However, it could be available from the corresponding author on reasonable request.
